# EXPLORING THE IMPACT OF TELE-GERIATRIC MENTAL HEALTH SERVICES ON RURAL CAREGIVERS’ WELL-BEING

**DOI:** 10.1093/geroni/igae098.1238

**Published:** 2024-12-31

**Authors:** Marika Humber, Chalise Carlson, Marisa-Francesca Brodrick, Rachel VanPutten, Amanda Peeples, Ranak Trivedi, Althea Lloyd, Christine Gould

**Affiliations:** VA Palo Alto Healthcare System, Palo Alto, California, United States; Department of Veterans Affairs, Palo Alto, California, United States; VA Palo Alto Healthcare System, Palo Alto, California, United States; VA Palo Alto Healthcare System, Palo Alto, California, United States; Corporal Michael J. Crescenz Department of Veterans Affairs Medical Center, Philadelphia, Pennsylvania, United States; Stanford University/VA Palo Alto Healthcare System, Palo Alto, California, United States; VISN 7 Clinical Resource Hub, VA Atlanta Health Care System, Decatur, Georgia, United States; VA Palo Alto Healthcare System, Palo Alto, California, United States

## Abstract

Rural caregivers may experience unique barriers to support including lack of services, transportation, and internet access. Within the Veterans Health Administration (VHA), a virtual geriatric psychiatry consultation service evolved to increase specialty mental health care access for rural older Veterans. Although the Veteran and their neurocognitive disorder (e.g., Alzheimer’s disease) and/or mental health condition (e.g., depression, PTSD) were the focus of the service, caregivers were also present at appointments. This study explored the impact of the service on rural caregivers in two VHA regions. The median age of patients was 77; caregivers were a spouse or adult child. Semi-structured interviews with caregivers (n=13) inquiring about overall experience and satisfaction with the service, changes to patient care and symptoms resulting from visits, and the service’s impact on the caregiver were conducted from September 2022-November 2023. Using rapid qualitative analysis, interview transcripts were summarized and compared to identify themes. Caregivers reported working with the geriatrics providers normalized the caregiving experience and provided helpful techniques and resources for managing challenging patient behaviors. Rural caregivers appreciated that visits were done at home alleviating the time and effort required to travel to distant VHA facilities and that providers could spend more time focusing on patient and caregiver needs. The direct access to specialty providers via follow-up appointments or by telephone to ask questions and address emergent problems was particularly valuable. These findings indicate that integrating caregivers into specialty tele-geriatric mental health services may decrease burden and improve well-being for caregivers of rural older Veterans.

